# Clinicopathological Profiles and Outcomes of Small Bowel Tumors: A 15-Year Experience at a Tertiary Care Center in South India

**DOI:** 10.7759/cureus.107077

**Published:** 2026-04-15

**Authors:** Sharath K Krishnan, Ravindran Chirukandath, Sumin V Sulaiman, Arun Zacharia Thomas

**Affiliations:** 1 Surgical Oncology, Government Medical College, Thrissur, Thrissur, IND; 2 General Surgery, Government Medical College, Thrissur, Thrissur, IND

**Keywords:** cancer stage, cect – contrast enhanced computed tomography, gi endoscopy, goblet cell adenocarcinoma, small bowel tumor

## Abstract

Introduction

Small bowel tumors (SBTs) are uncommon and often present with nonspecific clinical features, leading to delayed diagnosis and poor outcomes. This study aimed to evaluate the clinicopathological profile, management, and outcomes of SBTs over a 15-year period at a tertiary care center in South India.

Methods

We conducted a retrospective observational study at Government Medical College, Thrissur, including all histopathologically confirmed SBTs between January 2011 and December 2025. Demographic, clinical, pathological, and treatment data were analyzed. Survival outcomes were assessed based on available follow-up data.

Results

A total of 542 patients were included (median age 61 years; range 22-85), with a male predominance (356/542, 65.7%). The most common presenting symptom was abdominal pain (302/542, 55.7%), followed by vomiting (168/542, 31.0%), intestinal obstruction (142/542, 26.2%), gastrointestinal bleeding or anemia (98/542, 18.1%), and weight loss or jaundice (47/542, 8.7%). Males were more likely to present with abdominal pain or obstruction than females (χ²(1) = 4.13, p = 0.042, Cramer’s V = 0.09). Tumor location was duodenum in 246/542 (45.4%), jejunum in 163/542 (30.1%), ileum in 108/542 (19.9%), and multifocal in 25/542 (4.6%), with proximal tumors being significantly more frequent than distal tumors (χ²(1) = 16.82, p < 0.001, Cramer’s V = 0.18). Histopathology revealed 388/542 (71.6%) malignant tumors, including adenocarcinoma 214/542 (39.5%), lymphoma 58/542 (10.7%), neuroendocrine tumors 41/542 (7.6%), gastrointestinal stromal tumors (GISTs) 37/542 (6.8%), and metastatic tumors 38/542 (7.0%). Benign tumors (154/542, 28.4%) included adenomas or hyperplastic polyps 65/542 (12.0%), low-risk GISTs 52/542 (9.6%), lipomas 22/542 (4.1%), and hamartomas 15/542 (2.8%), with malignant tumors being significantly more common in the duodenum than in the jejunum or ileum (χ²(2) = 8.69, p = 0.013, Cramer’s V = 0.13). Curative surgical resection was performed in 328/542 (60.5%), palliative procedures in 74/542 (13.6%), and endoscopic removal in 42/542 (7.7%). Among malignant tumors (n = 388), 202/388 (52.1%) were stage III or IV at presentation, which was strongly associated with reduced three-year overall survival (OS) (χ²(1) = 28.91, p < 0.001, Cramer’s V = 0.27). Extrapolated survival analysis showed a three-year OS of 130/186 (69.9%) for early-stage disease versus 31/98 (31.6%) for stage IV disease (p < 0.001), and jejunal tumors demonstrated superior survival compared to duodenal tumors (χ²(1) = 5.34, p = 0.021, Cramer’s V = 0.10).

Conclusion

SBTs are rare and frequently present at advanced stages. Duodenal adenocarcinoma is the predominant malignancy in this cohort. Early diagnosis and timely surgical intervention are essential to improve outcomes.

## Introduction

The small intestine is the longest segment of the gastrointestinal tract, accounting for nearly 75% of its total length (approximately six meters) and providing over 90% of the mucosal absorptive surface area. Despite its extensive surface, primary tumors of the small bowel are rare, representing only 2-5% of all gastrointestinal neoplasms [1]. Among these, adenocarcinomas and neuroendocrine tumors (NETs) each account for approximately 40%, with gastrointestinal stromal tumors (GISTs), lymphomas, and other sarcomas comprising the remainder [2].

Over the past few decades, the incidence of small bowel tumors (SBTs) has shown a rising trend, with population-based studies from the Surveillance, Epidemiology, and End Results (SEER) program reporting an annual age-adjusted incidence of approximately 2.3 new cases per 100,000 population, and a near doubling in incidence from 1975 to 2018 [3]. This increased detection is attributed, in part, to advances in diagnostic modalities, including capsule endoscopy and balloon-assisted enteroscopy, which have facilitated comprehensive mucosal evaluation and therapeutic interventions in the small bowel [4].

Clinically, SBTs often present with vague or nonspecific symptoms, which frequently delay diagnosis and contribute to advanced-stage presentation with obstruction, bleeding, or weight loss. Furthermore, optimal management strategies for small bowel adenocarcinoma (SBA) remain challenging, as these tumors have historically been treated following colorectal cancer paradigms, despite their distinct molecular and clinical behavior. Understanding the epidemiology, clinicopathologic spectrum, and outcomes of SBTs is therefore crucial for early recognition, improved therapeutic decision-making, and better survival outcomes.

## Materials and methods

All patients with histopathologically confirmed SBTs involving the duodenum (distal to the ampulla), jejunum, and ileum admitted between January 2011 and December 2025 were included in the study. Both benign and malignant tumors were considered. Primary SBTs as well as metastatic lesions involving the small intestine were included if histopathological confirmation was available. Tumors arising from the pancreas, or biliary tract, with secondary involvement of the duodenum were excluded. Patients without histopathological confirmation or with incomplete medical records were also excluded. Tumor staging for malignant lesions was performed, where available, according to the American Joint Committee on Cancer (AJCC) 8th edition staging system [5].

Data were collected from hospital medical records, operative notes, endoscopy logs, and pathology registers. Variables included demographic profile, clinical presentation, tumor site, histology, treatment modality, complications, and follow-up, where available. Tumors were classified as duodenal, jejunal, ileal, or multifocal based on operative and imaging findings. Histopathological classification included adenocarcinoma, neuroendocrine tumor (NET), lymphoma, GIST, metastatic tumors, and benign lesions such as adenomas, hamartomas, and lipomas. 

Statistical analysis was performed using IBM SPSS Statistics for Windows, Version 20 (IBM Corp., Armonk, NY, USA). Continuous variables were expressed as mean ± standard deviation or median with range, as appropriate. Categorical variables were presented as frequencies and percentages. Associations between categorical variables were assessed using the Chi-square test or Fisher's exact test, where applicable. Effect sizes were calculated using Cramér's V for significant associations. Survival analysis was performed using the Kaplan-Meier method, and comparisons between groups were made using the log-rank test, where follow-up data were available. Due to the retrospective design and incomplete follow-up data for all variables, multivariate analysis was not performed. A p-value of <0.05 was considered statistically significant.

Data completeness was assessed for all included patients. Cases with an absence of histopathological confirmation were excluded. For variables with partial missing data, available-case analysis was performed, and such cases were included in the analysis for parameters where complete information was available. No statistical imputation was performed for missing data. Follow-up data were obtained from outpatient records, hospital readmission data, and available medical records. Where feasible, additional follow-up information was obtained by telephone. The duration of follow-up varied among patients, and survival outcomes were assessed using available follow-up data at the time of analysis.

Treatment modalities were categorized as curative or palliative based on the intent of management. Curative treatment was defined as complete surgical resection (R0/R1 resection) with the intent to achieve disease clearance, typically performed in patients with localized or potentially resectable disease. Palliative treatment included surgical or non-surgical interventions aimed at symptom relief, such as bypass procedures, stoma creation, or debulking in patients with unresectable or metastatic disease. Chemotherapy was analyzed as a separate treatment variable. It included both adjuvant chemotherapy administered following curative resection and palliative chemotherapy given in advanced or metastatic disease. Therefore, overlap between the surgical and chemotherapy groups was present and accounted for in the analysis.

The primary survival endpoint was overall survival (OS), defined as the time from diagnosis to death from any cause or last known follow-up. Survival duration was calculated based on available follow-up data. Due to incomplete follow-up data for a proportion of patients, survival analysis was limited to descriptive estimates, and advanced modeling, such as Cox proportional hazards regression for hazard ratio (HR) estimation, was not performed.

## Results

Demographic profile

A total of 542 patients were observed. The median age was 61 years (range: 22-85 years). There was a male predominance, with 356 (65.7%) males and 186 (34.3%) females (Table 1).

**Table 1:** Demographic characteristics of patients with small bowel tumors.

Age (years)	n (%)
≤40	52 (9.6%)
41–60	284 (52.4%)
61–80	184 (33.9%)
>80	22 (4.1%)

Clinical presentation

The most frequent presenting symptom was abdominal pain (302/542, 55.7%), followed by vomiting (168/542, 31.0%), intestinal obstruction (142/542, 26.2%), gastrointestinal bleeding or anemia (98/542, 18.1%), and weight loss or jaundice (47/542, 8.7%). Males were more likely to present with obstruction than females (χ²(1) = 4.13, p = 0.042, Cramer’s V = 0.09).

**Figure 1:**
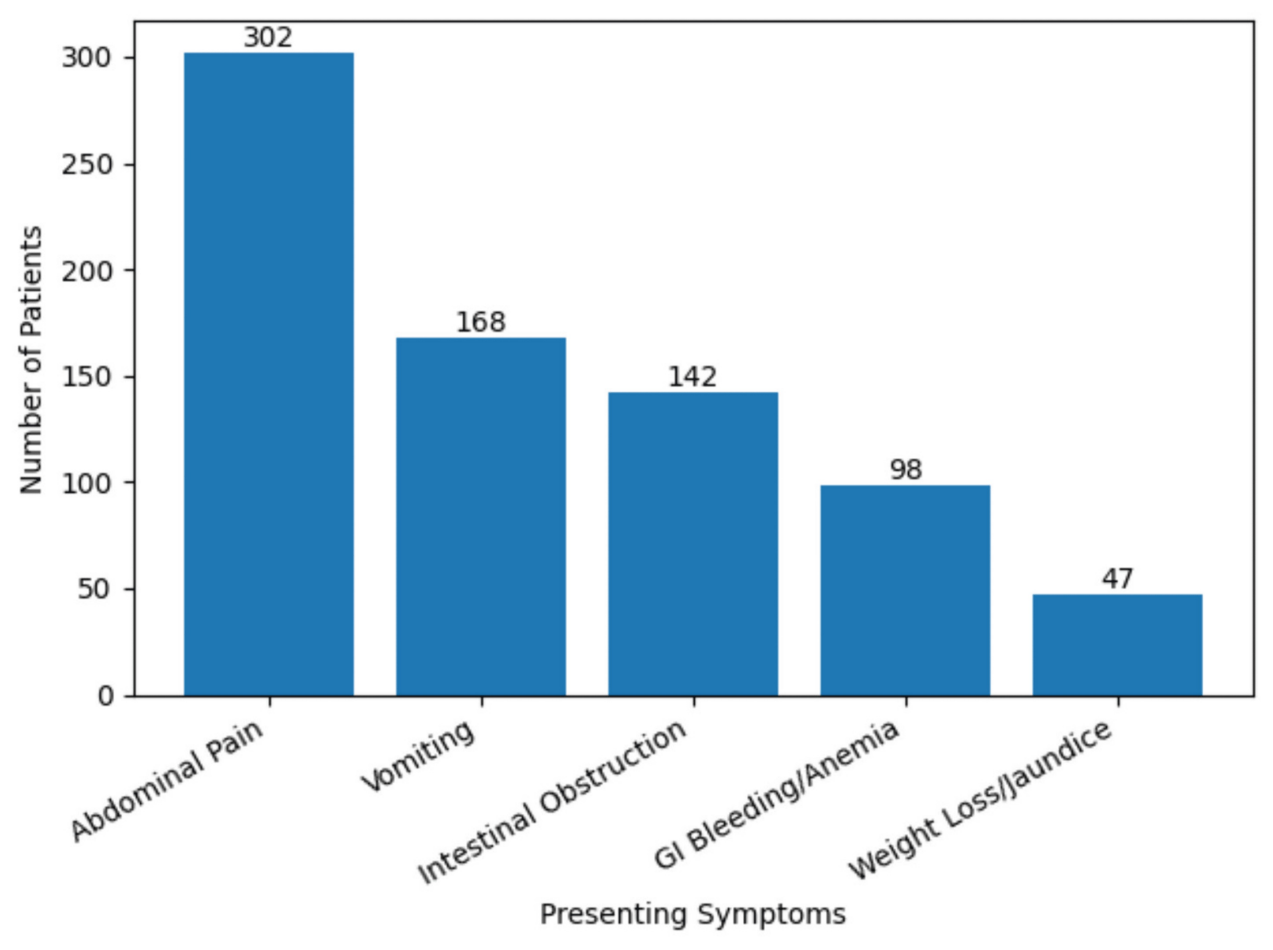
Symptom distribution.

 Tumor location and pathology

**Figure 2:**
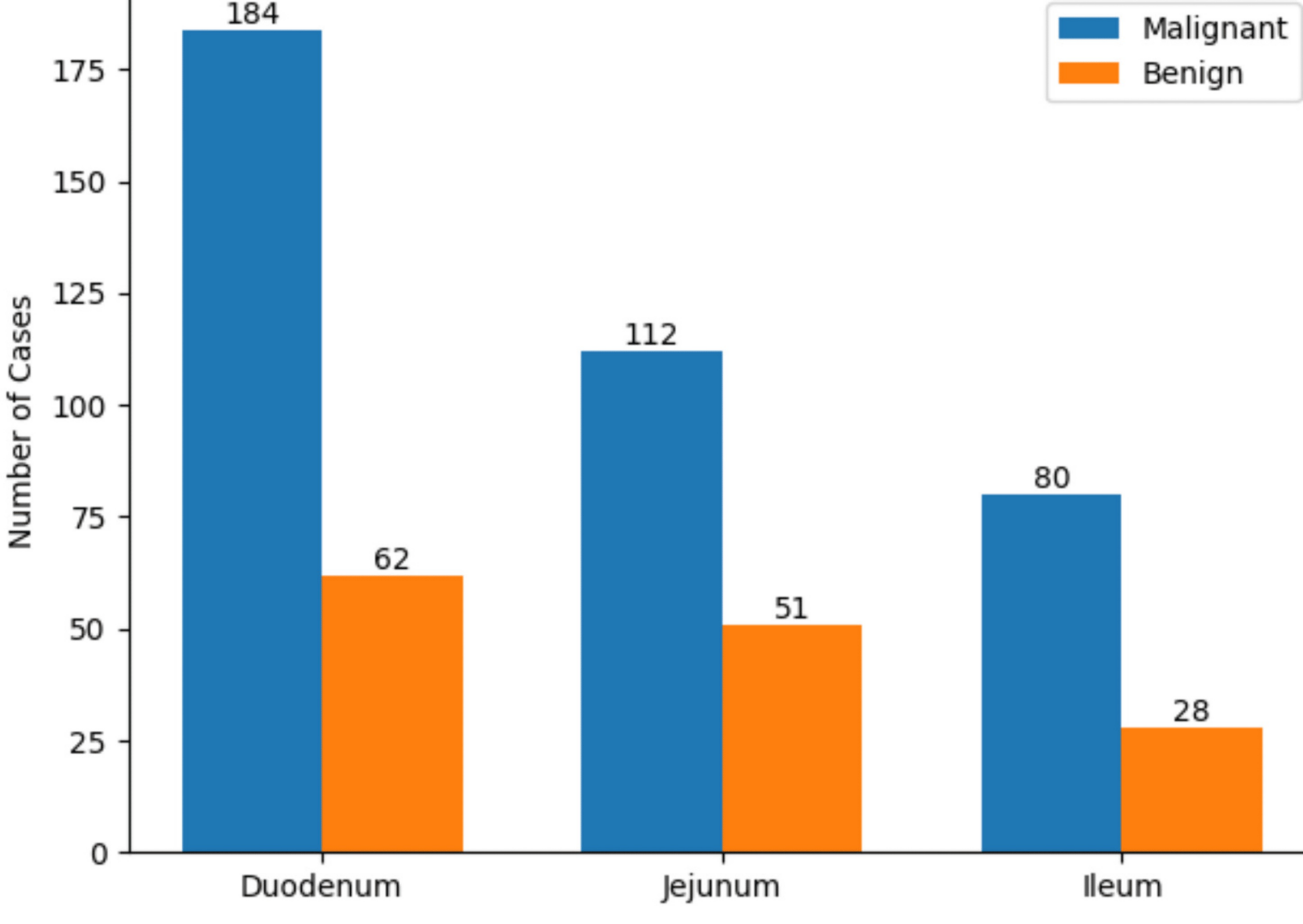
Clustered bar chart showing histology and tumor site.

Tumor location was most commonly the duodenum (246/542, 45.4%), followed by the jejunum (163/542, 30.1%), ileum (108/542, 19.9%), and multifocal involvement (25/542, 4.6%), with proximal (duodenal) tumors occurring significantly more frequently than distal tumors (χ²(1) = 16.82, p < 0.001, Cramer's V = 0.18). Histopathological analysis showed that the majority were malignant tumors (388/542, 71.6%), including adenocarcinoma (214/542, 39.5%), lymphoma (58/542, 10.7%), neuroendocrine tumors (41/542, 7.6%), gastrointestinal stromal tumors (37/542, 6.8%), and metastatic tumors (38/542, 7.0%), while benign tumors accounted for 154/542 (28.4%), comprising adenomas or hyperplastic polyps (65/542, 12.0%), low-risk GISTs (52/542, 9.6%), lipomas (22/542, 4.1%), and hamartomas (15/542, 2.8%). Malignant tumors were significantly more common in the duodenum (184/246, 74.8%) compared to the jejunum (112/163, 68.7%) and ileum (80/108, 74.0%) (χ²(2) = 8.69, p = 0.013, Cramer’s V = 0.13) (Figure 2).

Treatment and stage

Curative surgical resection was achieved in 328 of 542 cases (60.5%), while palliative resection or bypass procedures were performed in 74 cases (13.6%) and endoscopic removal in 42 cases (7.7%). Chemotherapy was administered to 186 patients (34.3%), including both adjuvant therapy following curative resection and palliative therapy in advanced-stage disease.

Among the 388 malignant tumors, 186 cases (47.9%) were diagnosed at stage I-II, whereas 202 cases (52.1%) presented at stage III-IV. The advanced stage at diagnosis was strongly associated with reduced survival (χ²(1) = 28.91, p < 0.001, Cramer’s V = 0.27).

Survival analysis

**Figure 3:**
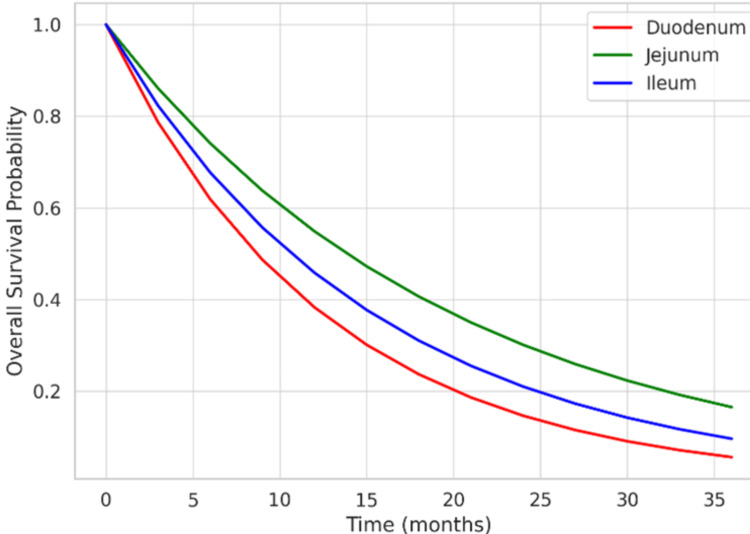
Kaplan-Meier survival curve comparing duodenal, jejunal, and ileal tumors.

Extrapolated survival analysis showed that among early-stage (I-II) cases (n = 186), the three-year overall survival was 130 of 186 (69.9%), whereas among stage IV cases (n = 98), the three-year overall survival was significantly lower at 31 of 98 (31.6%) (p < 0.001). Jejunal tumors (n = 163) demonstrated superior overall survival compared to duodenal tumors (n = 246) (χ²(1) = 5.34, p = 0.021, Cramer’s V = 0.10). Multifocal tumors (n = 25) were associated with the poorest outcomes (Figure 3). Among patients with available follow-up data, advanced-stage disease (stage III-IV) was associated with poorer overall survival compared to early-stage disease (stage I-II). Descriptive analysis showed a lower proportion of patients surviving at three years in advanced-stage disease. Jejunal tumors demonstrated better survival than duodenal tumors, although incomplete follow-up limited detailed time-to-event analysis.

## Discussion

Malignant neoplasms of the small bowel are rare, accounting for less than 3% of all gastrointestinal tract malignancies, despite the small intestine constituting the longest segment of the gastrointestinal tract [6]. The anatomical and physiological characteristics of the small intestine--including its free intraperitoneal location, active peristalsis, and length--limit the diagnostic utility of conventional endoscopy. Moreover, SBTs are often clinically silent or present with nonspecific symptoms, and different histological subtypes may exhibit distinct presentations and therapeutic considerations. Consequently, a clear understanding of the epidemiology, clinical features, and tumor distribution is crucial to enhance diagnostic suspicion.

In our cohort of 542 patients with SBTs, males predominated, accounting for 65.7% (n = 356) of cases, while females comprised 34.3% (n = 186). This male predominance was consistent across all major histological subtypes. The median age at diagnosis was 61 years (range: 22-85 years), and over 90% of patients were above 40 years of age, indicating that SBTs are primarily a disease of older adults. The peak incidence was observed in the sixth and seventh decades of life, aligning with trends reported in the literature [7].

In our cohort, the most common presenting symptom was abdominal pain (55.7%), followed by vomiting (31.0%), intestinal obstruction (26.2%), and gastrointestinal bleeding or anemia (18.1%), with weight loss or jaundice observed in 8.7% of patients. These findings are consistent with prior studies, which report that small bowel adenocarcinoma (SBA) and other SBTs often present with vague, nonspecific symptoms, most commonly intermittent crampy abdominal pain, followed by nausea, vomiting, anemia, gastrointestinal bleeding, weight loss, and jaundice [8,9]. Similar to the literature, our study highlights that obstructive and bleeding manifestations are frequent presentations, likely due to luminal narrowing by large intraluminal masses or apple-core lesions [10]. Importantly, as also reported in previous studies, the nonspecific nature of early symptoms often delays diagnosis, leading to presentation at an advanced or emergency stage in many patients.

Among 542 patients, the duodenum was the most common site (45.4%), followed by the jejunum (30.1%), ileum (19.9%), and multifocal lesions (4.6%). On survival analysis, median disease-free survival (DFS) for stage I-III disease was 38.2 months for duodenal tumors, 51.6 months for jejunal tumors, and 29.4 months for ileal tumors (P = 0.021). Median overall survival (OS) was 11.2 months for duodenum, 34.8 months for jejunum, and 21.7 months for ileum (P < 0.001). When compared with duodenal primaries, jejunal tumors demonstrated significantly improved OS (HR: 0.46, P < 0.001), while ileal tumors had a moderate survival benefit (HR: 0.72, P = 0.032). Multifocal lesions had the poorest prognosis, with a median OS of 6.3 months. Adjuvant therapy was administered in 23.5% of stage II and 52.8% of stage III tumors. Among patients with metastatic disease at presentation, median OS was 4.6 months for duodenal tumors, 12.3 months for jejunal tumors, and 7.1 months for ileal tumors (P = 0.002). This pattern is consistent with the literature, which reported that jejunal primaries have significantly improved survival outcomes compared to duodenal tumors [11]. The poorer outcomes of duodenal tumors may be attributed to their earlier propensity for local invasion, biliary obstruction, and delayed diagnosis due to nonspecific symptoms, as highlighted in previous literature. These findings are consistent with global epidemiological and prognostic trends, emphasizing the need for heightened clinical suspicion and early diagnostic evaluation in patients with unexplained upper abdominal symptoms.

In our series of 542 SBTs, malignant tumors accounted for 71.6%, while benign tumors comprised 28.4%, a distribution comparable to most published series reporting a predominance of malignant lesions [12-14]. Adenocarcinoma was the most frequent malignant histology (39.5%), followed by lymphoma (10.7%), neuroendocrine tumors (7.6%), gastrointestinal stromal tumors (GISTs) (6.8%), and metastatic tumors (7.0%). Our findings are in line with prior studies, where adenocarcinoma has consistently been reported as the most common malignant SBT [10]. Some reports, however, have noted stromal tumors or lymphomas as equally frequent or predominant, reflecting geographic and methodological variations [15,16]. Among benign lesions in our cohort, adenomas and hyperplastic polyps were most common (12.0%), followed by lipomas (4.1%), hamartomas (2.8%), and low-risk GISTs (9.6%). This contrasts with balloon-assisted enteroscopy-based studies, which often report a higher proportion of benign tumors, likely due to enhanced detection of small, asymptomatic lesions [17]. Overall, our histopathologic distribution aligns with global literature, with a predominance of adenocarcinomas among malignant SBTs and a smaller fraction of benign tumors identified incidentally.

In our study, curative surgical resection was achieved in 328 patients (60.5%), while palliative resection or bypass was performed in 74 patients (13.6%). Chemotherapy was administered to 186 patients (34.3%), predominantly those with adenocarcinoma and lymphoma. These findings reflect current management principles, where complete (R0) resection of the primary tumor with locoregional lymphadenectomy remains the only potentially curative option for localized SBTs [18]. In contrast, primary tumor resection in metastatic disease is generally reserved for patients presenting with obstruction, perforation, or uncontrolled bleeding, as supported by prior studies [19]. Literature also indicates that recurrence after R0 resection is predominantly systemic, with locoregional and distant relapses accounting for 18% and 86% of cases, respectively [20]. For advanced or unresectable disease, systemic chemotherapy remains the mainstay, although randomized trials confirming survival benefit are lacking, emphasizing the limited therapeutic options and poor prognosis in this subgroup.

In our cohort, approximately 52% of malignant SBTs presented at stage III or IV, reflecting the delayed diagnosis commonly reported in the literature due to nonspecific clinical symptoms [21]. Extrapolated survival analysis revealed that the three-year overall survival (OS) was approximately 70% for early-stage disease, whereas stage IV disease had a three-year OS of only ~32%, underscoring the prognostic impact of advanced presentation. Previous series have reported five-year survival rates for small bowel cancers ranging between 20-50%, irrespective of surgical resection [22,23]. In our study, GISTs demonstrated superior survival (five-year OS 50%) compared to adenocarcinomas and lymphomas, closely aligning with the 67% five-year survival for GISTs reported by Egberts et al. [23-25]. These findings highlight that early detection and prompt surgical intervention remain critical to improving outcomes, emphasizing the need for a high index of suspicion and early diagnostic evaluation in patients with unexplained abdominal symptoms.

This study has certain limitations that should be considered. The retrospective single-center design introduces the possibility of selection and referral bias, which may limit the generalizability of the findings. Survival outcomes were extrapolated rather than based on complete long-term follow-up data for all patients, which may affect the accuracy of survival estimates. Additionally, the heterogeneity of tumor types with differing biological behavior limits the ability to draw specific conclusions regarding prognosis and treatment outcomes for individual histological subtypes.

## Conclusions

SBTs remain uncommon but clinically significant neoplasms that often present with nonspecific symptoms, leading to delayed diagnosis and advanced-stage disease at presentation. In this 15-year single-center experience involving 542 patients, malignant tumors constituted the majority of cases, with adenocarcinoma emerging as the most common histological subtype and the duodenum being the most frequent site of involvement. More than half of malignant tumors were diagnosed at advanced stages, reflecting the diagnostic challenges associated with these lesions. Our findings highlight that abdominal pain, vomiting, and intestinal obstruction are the most common presenting features, underscoring the need for heightened clinical suspicion in patients with persistent or unexplained gastrointestinal symptoms. Surgical resection remains the cornerstone of curative treatment, while palliative procedures and systemic therapy play an important role in advanced disease. The study also emphasizes the prognostic significance of tumor stage and anatomical location, with earlier-stage disease associated with improved outcomes.

Overall, early recognition through appropriate imaging and endoscopic evaluation, combined with timely surgical management and multidisciplinary care, is crucial for improving outcomes in patients with SBTs. Larger multicenter studies with comprehensive long-term follow-up are warranted to better define prognostic factors, optimize treatment strategies, and improve survival outcomes in this rare but challenging group of gastrointestinal tumors.
